# The Triglyceride-Glucose Index as a Biomarker for Insulin Resistance Following Hepatitis C Virus Eradication: A Prospective Cohort Study

**DOI:** 10.3390/jcm14092963

**Published:** 2025-04-25

**Authors:** Shih-Hsiung Shen, Hsin-Ju Tsai, Yu-Hsuan Li, Chia-Chang Chen, Ying-Cheng Lin, Shou-Wu Lee, Sheng-Shun Yang, Yi-Hsiang Huang, Teng-Yu Lee

**Affiliations:** 1 Division of Gastroenterology and Hepatology, Department of Internal Medicine, Taichung Veterans General Hospital, Taichung 407219, Taiwanethankevin516@vghtc.gov.tw (Y.-C.L.);; 2 Department of Post-Baccalaureate Medicine, College of Medicine, National Chung Hsing University, Taichung 40227, Taiwan; 3 Department of Computer Science & Information Engineering, National Taiwan University, Taipei 10617, Taiwan; brightlight@vghtc.gov.tw; 4 Division of Endocrinology and Metabolism, Department of Internal Medicine, Taichung Veterans General Hospital, Taichung 407219, Taiwan; 5 School of Medicine, Chung Shan Medical University, Taichung 40201, Taiwan; 6 Ph.D. Program in Translational Medicine, National Chung Hsing University, Taichung 40227, Taiwan; 7 Institute of Biomedical Sciences, National Chung Hsing University, Taichung 40227, Taiwan; 8 Division of Gastroenterology and Hepatology, Department of Medicine, Taipei Veterans General Hospital, Taipei 11217, Taiwan; 9 Institute of Clinical Medicine, National Yang Ming Chiao Tung University, Taipei 11230, Taiwan

**Keywords:** hepatitis C virus, direct-acting antivirals, HOMA-IR, insulin resistance, TyG index

## Abstract

**Background:** The triglyceride-glucose (TyG) index has emerged as a novel surrogate marker of insulin resistance, but its changes after hepatitis C virus (HCV) eradication remain unclear. This study aimed to evaluate changes in the TyG index following direct-acting antiviral (DAA) treatment. **Methods:** HCV-infected patients achieving sustained virological response 12 weeks post-treatment (SVR12) were prospectively enrolled from May 2015 to June 2023. Exclusion criteria included the following: (1) failure to achieve SVR12; (2) use of anti-diabetes or anti-hyperlipidemia medications; and (3) hepatitis B virus or human immunodeficiency virus co-infection. Changes in lipid profiles, TyG index, and homeostasis model assessment of insulin resistance (HOMA-IR) were evaluated from baseline to SVR12. Insulin resistance was defined as HOMA-IR ≥ 2.5. The optimal TyG index cut-off for predicting insulin resistance was determined using the Youden Index. **Results:** A total of 111 patients (median age: 61.0 years; 45.9% male) were included. The TyG index correlated positively with HOMA-IR (Pearson’s *r* = 0.32, *p* < 0.001). Among patients with pre-existing insulin resistance, significant improvements were observed at SVR12 in both HOMA-IR (4.0 [IQR: 3.1–5.4] vs. 2.5 [IQR: 2.0–3.9]; *p* < 0.001) and TyG index (8.47 [IQR: 8.08–8.68] vs. 8.36 [IQR: 8.00–8.71]; *p* = 0.028). Using 8.27 as the optimal TyG index cut-off, similar improvements were noted in HOMA-IR (2.8 [IQR: 2.0–4.3] vs. 2.3 [IQR: 1.5–3.8]; *p* = 0.031) and TyG index (8.62 [IQR: 8.46–8.83] vs. 8.52 [IQR: 8.27–8.89]; *p* = 0.003). **Conclusions:** The TyG index is a valuable tool for monitoring changes in insulin resistance after HCV eradication, particularly in patients with baseline insulin resistance.

## 1. Introduction

Hepatitis C virus (HCV) infection leads to liver-related complications, such as cirrhosis and hepatocellular carcinoma, both of which contribute significantly to the public health burden [1,2]. In addition, HCV infection is associated with host metabolic dysfunction, and the prevalence of insulin resistance and type 2 diabetes mellitus (DM) is higher in HCV-infected patients compared to the general population [3]. Importantly, increased insulin resistance in HCV-infected individuals further raises the risk of adverse outcomes, not only liver-related diseases but also extrahepatic conditions, such as renal disease and cardiovascular events. Successful HCV eradication through antiviral therapy has been shown to improve insulin resistance, as well as reduce the risk of extrahepatic complications associated with HCV [4,5]. In recent years, the development of direct-acting antivirals (DAAs) has made HCV eradication more achievable, with evidence suggesting that DAAs also improve insulin resistance in HCV-infected patients [6,7,8,9,10].

However, even after successful HCV eradication, some patients may continue to experience insulin resistance, which remains one of the most significant risk factors for the development of hepatocellular carcinoma (HCC) [11]. For personalized surveillance programs following DAA therapy, a practical and reliable tool for accurately assessing insulin resistance is essential. The gold standard for evaluating insulin resistance is the hyperinsulinemic-euglycemic clamp (HIEC) test, but this method is not feasible for large population-based studies or routine clinical practice due to its complexity and high cost [12,13]. The homeostasis model assessment of insulin resistance (HOMA-IR) is the most widely used surrogate marker of insulin resistance, but it has limited applicability in individuals receiving insulin treatment [14,15]. Furthermore, because circulating insulin levels are not routinely measured, the use of HOMA-IR is further constrained [12]. In contrast, the triglyceride-glucose (TyG) index is a simple, accessible, and reliable surrogate marker of insulin resistance [16]. The diagnostic accuracy of the TyG index for identifying insulin resistance, using HIEC and HOMA-IR as reference methods, has been validated in several studies. The TyG index has shown strong performance in estimating insulin resistance, both in individuals with and without diabetes [17]. Although several studies have demonstrated that insulin resistance in HCV-infected patients improves with DAA therapy using HOMA-IR, there remains a lack of data regarding changes in the TyG index after HCV eradication. Therefore, we aim to conduct a prospective cohort study to compare changes in the TyG index and HOMA-IR following HCV eradication through DAA treatment in patients with chronic HCV infection.

## 2. Patients and Methods

### 2.1. Study Design

This cohort study was conducted at Taichung Veterans General Hospital, a tertiary medical center located in central Taiwan. Study subjects were prospectively enrolled from 1 May 2015 to 30 June 2023 and were followed up for 12 weeks post-treatment to assess sustained virological response at 12 weeks (SVR12). The follow-up deadline was 31 December 2023. This study was approved by the Institutional Review Board of Taichung Veterans General Hospital (CF13095, 26 March 2013). All participants provided written informed consent prior to enrollment and before any study procedures commenced. The study design, data collection, and analysis adhered to the Declaration of Helsinki.

### 2.2. Study Subjects

The inclusion criteria were as follows: (1) ≥18 years of age; (2) chronic hepatitis C with HCV viremia; and (3) indicated for DAA therapy (according to the Taiwan practice guidelines, DAA therapy is recommended for all HCV-viremic patients with a life expectancy of ≥1 year) [18]. The exclusion criteria were as follows: (1) not achieving SVR12 after DAA treatment; (2) DM defined as fasting plasma glucose ≥ 126 mg/dL, percentage of glycosylated hemoglobin (HbA1c) ≥ 6.5% and/or under treatment for diabetes [19]; (3) under anti-hyperlipidemia medications; (4) hepatitis B virus or human immunodeficiency virus co-infection; (5) pregnancy; or (6) incomplete study data collection.

### 2.3. Main Outcome Measurements

The main outcome was the change in insulin resistance following HCV eradication. Both HOMA-IR and TyG index were assessed at baseline and 12 weeks post-DAA treatment. The HOMA-IR was calculated by the following formula: (fasting plasma insulin [μU/mL] × fasting plasma glucose [mg/dL])/405 [14]. The TyG index was calculated using the following formula: Ln (fasting triglycerides [mg/dL] × fasting plasma glucose [mg/dL]/2) [16]. In this study, patients with a HOMA-IR score ≥ 2.5 were defined as insulin resistance [16,20].

### 2.4. Hepatic and Metabolic Parameter Measurements

Hepatic parameters, including aspartate aminotransferase (AST), alanine aminotransferase (ALT), and baseline HCV viral load, were all collected. The AST-to-platelet ratio index (APRI) and Fibrosis-4 (FIB-4) index were used to access the degree of liver fibrosis [21,22]. The APRI score was calculated as follows: ([AST/upper limit of the normal AST range] × 100)/Platelet Count. The FIB-4 score was determined according to the formula: (Age × AST)/(Platelet count × √ALT). Cutoff values of <1.45, 1.45–3.25, and >3.25 were used to assess the degree of liver fibrosis [23]. Liver cirrhosis is primarily diagnosed through an abdominal sonogram showing the coarsening of the parenchyma, as well as splenomegaly, which is defined as a spleen longer than 12 cm [24]. Hepatic steatosis was assessed by abdominal sonogram. The grading of steatosis (mild, moderate, or severe) was based on standard criteria, including increased hepatic echogenicity, the discrepancy between hepatic and renal cortex echoes, and reduced visualization of portal vein walls structures [25]. Lipid profiles, including total cholesterol, high-density lipoprotein (HDL) cholesterol, low-density lipoprotein (LDL), and triglyceride, were all examined after fasting for 8 h or more. Fasting plasma glucose and glycosylated hemoglobin (HbA1c) were also checked. Patients with prediabetes were defined according to the American Diabetes Association criteria, as having impaired fasting glucose (fasting plasma glucose between 100 and 125 mg/dL) and/or an HbA1c level between 5.7% and 6.4% [26].

### 2.5. Statistical Analyses

Categorical variables were presented as both numbers and percentages, while continuous variables were reported as a median with interquartile ranges (IQRs). Continuous variables were compared through use of the Wilcoxon Signed-Rank test, where two-tailed *p* values < 0.05 were considered significant. The correlation between the TyG index and HOMA-IR was computed using the Pearson correlation coefficient formula. The optimal TyG index for estimating insulin resistance was determined through receiver operating characteristic (ROC) curve analysis. All data were managed and analyzed using SPSS version 24 (IBM Armonk, New York, NY, USA).

## 3. Results

### 3.1. Baseline Characteristics

A total of 111 patients were included in the final analysis. As indicated in Table 1, the median age was 61.0 years, and male patients accounted for 45.9% of the cohort. About 18.0% of the patients were diagnosed with underlying liver cirrhosis prior to receiving DAA treatment. The median values of baseline body mass index, total cholesterol, HDL cholesterol, LDL cholesterol, triglyceride, fasting plasma sugar, and HbA1c were all within normal ranges. The median HOMA-IR was 2.4 (1.4–3.9), in which 53 (47.7%) patients were diagnosed with baseline insulin resistance, i.e., HOMA-IR ≥ 2.5.

### 3.2. The Correlations Between HOMA-IR and TyG Index

**Figure 1:**
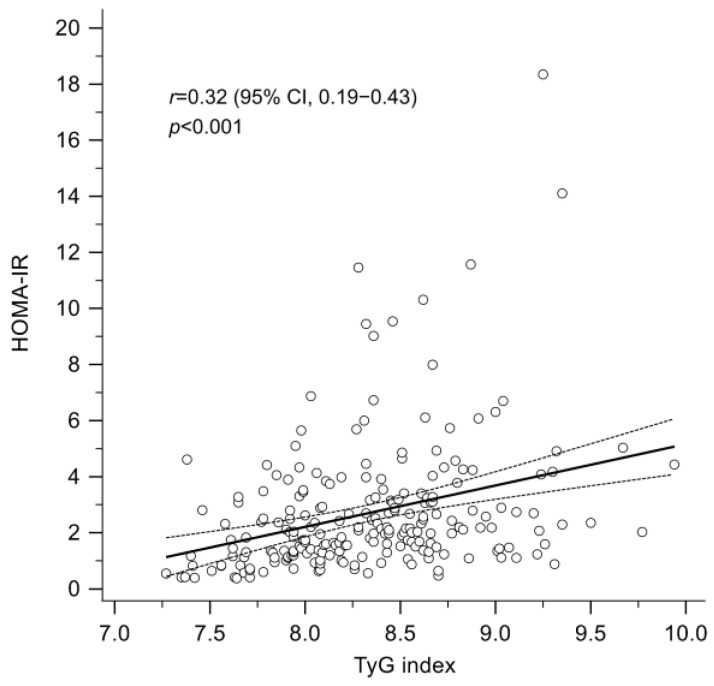
A significant positive correlation between the TyG index and the HOMA-IR (Pearson’s correlation coefficient, *r* = 0.32; *p* < 0.001). HOMA-IR, homeostasis model assessment index for insulin resistance; TyG index, triglyceride-glucose index.

As shown in Figure 1, there was a significant positive correlation between the TyG index and HOMA-IR (Pearson’s *r* = 0.32; *p* < 0.001). Using HOMA-IR ≥ 2.5 as the definition of insulin resistance, the optimal cut-off value of the TyG index to predict insulin resistance was determined using the Youden Index, and the diagnostic performance was evaluated through area under the ROC curve (AUC) analysis. As depicted in Supplemental Figure S1, with 8.27 identified as the optimal cut-off value for the TyG index, the AUC for insulin resistance was 0.63 (95% confidence interval: 0.57–0.70; *p* < 0.001), with a sensitivity of 68.6% and a specificity of 57.4%. The positive and negative predictive values were 59.7% and 67.3%, respectively. A total of 62 patients (55.9%) had a baseline TyG index ≥ 8.27.

### 3.3. Metabolic Changes in Patients with Insulin Resistance

**Figure 2:**
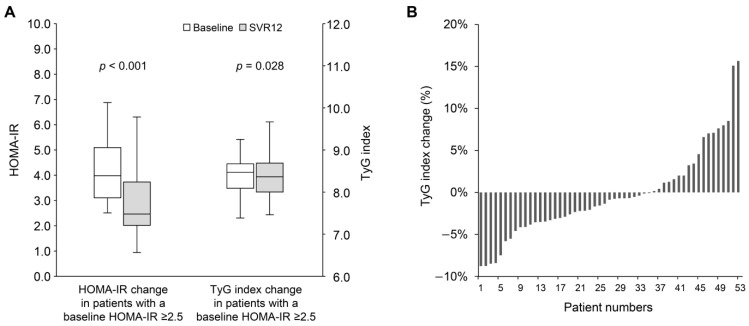
(**A**) The change in HOMA-IR and TyG index from baseline to SVR12 in patients with baseline HOMA-IR ≥ 2.5. (**B**) The percentage change in TyG index from baseline to SVR12 in patients with baseline HOMA-IR ≥ 2.5. HOMA-IR, homeostasis model assessment index for insulin resistance; SVR12, sustained virological response at 12 weeks post-treatment; TyG index, triglyceride-glucose index.

Table 2 shows changes in hepatic and metabolic parameters in patients with impaired insulin resistance, defined by a baseline HOMA-IR ≥ 2.5. Notable improvements were observed in hepatic parameters, including AST, ALT, and APRI. The median values of total cholesterol (158.0 [IQR: 140.0–184.5] vs. 179.0 [IQR: 153.0–203.0] mg/dL; *p* = 0.004) and LDL cholesterol (92.0 [IQR: 75.5–108.5] vs. 112.0 [IQR: 90.5–123.5] mg/dL; *p* = 0.006) showed significant increases. Although a decrease in triglycerides was observed, it was not statistically significant (101.0 [IQR: 65.0–125.5] vs. 87.0 [IQR: 63.5–123.0] mg/dL; *p* = 0.272). The median values of HbA1c remained stable (5.5 [IQR: 5.3–5.8] vs. 5.5 [IQR: 5.3–5.8] %; *p* = 1.000), while fasting plasma glucose significantly declined (100.0 [IQR: 96.0–109.0] vs. 100.0 [IQR: 90.0–109.0] mg/dL; *p* = 0.028). Furthermore, the median insulin level significantly decreased (15.4 [IQR: 12.4–23.9] vs. 10.3 [IQR: 7.8–16.2] mg/dL; *p* < 0.001). A significant correlation was observed between the baseline HOMA-IR and the change in HOMA-IR at SVR 12 (Pearson’s *r* = −0.55; *p* < 0.001) (Supplemental Figure S2). In patients with a baseline HOMA-IR ≥ 2.5, exhibited a significant improvement in both HOMA-IR (4.0 [IQR: 3.1–5.4] vs. 2.5 [IQR: 2.0–3.9]; *p* < 0.001) and TyG index (8.47 [IQR: 8.08–8.68] vs. 8.36 [IQR: 8.00–8.71]; *p* = 0.028) (Figure 2A). Among them, 35 patients (66.0%) demonstrated an improvement in TyG index at SVR12 (Figure 2B), In contrast, patients with a baseline HOMA-IR of less than 2.5 did not show significant changes in HOMA-IR (1.4 [IQR: 1.0–2.0] vs. 1.3 [IQR: 0.9–2.0]; *p* = 0.896) or TyG index (8.20 [IQR: 7.88–8.57] vs. 8.09 [IQR: 7.90–8.57]; *p* = 1.000).

### 3.4. Metabolic Changes in Patients with a TyG Index ≥ 8.27

**Figure 3:**
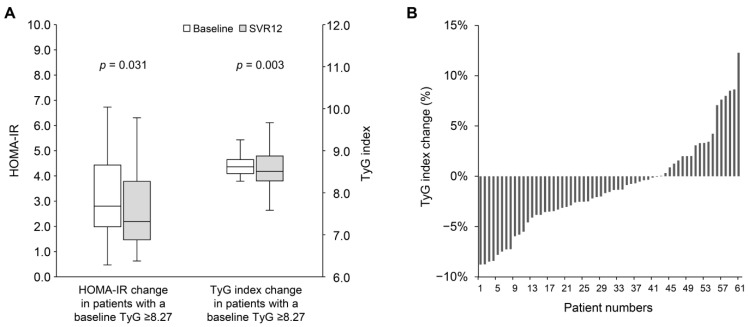
(**A**) The change in HOMA-IR and TyG index from baseline to SVR12 in patients with baseline TyG index ≥ 8.27. (**B**) The percentage change in TyG index from baseline to SVR12 in patients with baseline TyG index ≥ 8.27. HOMA-IR, homeostasis model assessment index for insulin resistance; SVR12, sustained virological response at 12 weeks post-treatment; TyG index, triglyceride-glucose index.

As shown in Table 3, in patients with a baseline TyG index ≥ 8.27, the median levels of total cholesterol (160.0 [IQR: 146.0–184.3] vs. 179.0 [IQR: 159.8–203.0] mg/dL; *p* < 0.001) and LDL cholesterol (93.0 [IQR: 78.8–111.5] vs. 111.5 [IQR: 94.8–125.3] mg/dL; *p* < 0.001) increased significantly. Interestingly, triglyceride levels decreased significantly (112.0 [IQR: 100.3–137.8] vs. 99.5 [IQR: 70.8–139.5] mg/dL; *p* = 0.001). However, no significant changes were observed in median HbA1c (5.5 [IQR: 5.2–5.9] vs. 5.6 [IQR: 5.3–5.8] %; *p* = 0.272) or fasting plasma glucose (99.0 [IQR: 91.5–108.0] vs. 99.5 [IQR: 93.0–109.0] mg/dL; *p* = 0.699). The median insulin level decreased significantly (11.3 [IQR: 8.2–16.4] vs. 9.1 [IQR: 6.1–15.9] mg/dL; *p* = 0.010). Additionally, there was a significant correlation between the baseline TyG index and its change at SVR12 (Pearson’s *r* = −0.35; *p* < 0.001) (Supplemental Figure S3). Patients with a baseline TyG index ≥ 8.27 experienced significant improvements in both HOMA-IR (2.8 [IQR: 2.0–4.3] vs. 2.3 [IQR: 1.5–3.8]; *p* = 0.031) and TyG index (8.62 [IQR: 8.46–8.83] vs. 8.52 [IQR: 8.27–8.89]; *p* = 0.003) (Figure 3A). Among these patients, 43 patients (68.3%) showed improvement in the TyG index at SVR12 (Figure 3B). Conversely, patients with a baseline TyG index < 8.27 exhibited a modest but significant improvement in HOMA-IR (1.6 [IQR: 1.0–3.5] vs. 1.6 [IQR: 0.9–2.2]; *p* = 0.046), while no significant change was observed in the TyG index (7.95 [IQR: 7.69–8.12] vs. 7.97 [IQR: 7.79–8.24]; *p* = 0.312).

### 3.5. Metabolic Changes in Patients with Prediabetes

**Figure 4:**
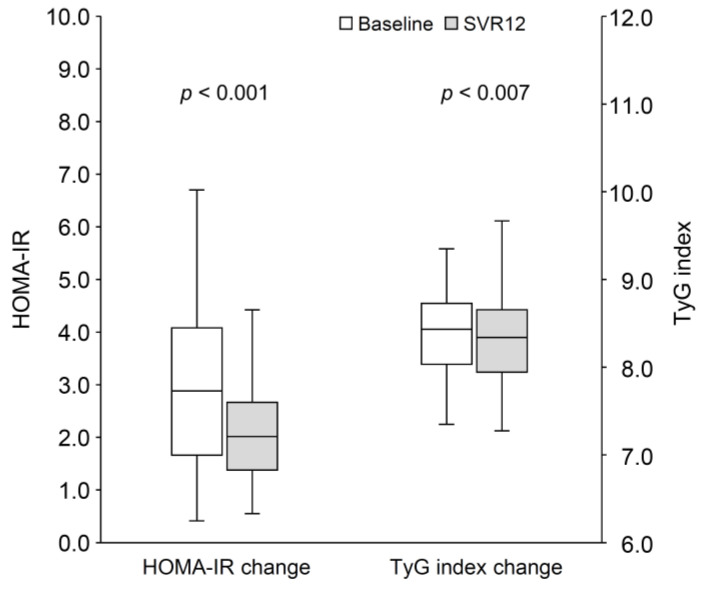
The change in HOMA-IR and TyG index from baseline to SVR12 in patients with prediabetes. HOMA-IR, homeostasis model assessment index for insulin resistance; SVR12, sustained virological response at 12 weeks post-treatment; TyG index, triglyceride-glucose index.

As shown in Table 4, 59 patients (53.2%) were classified as having prediabetes. The trend of increasing cholesterol from baseline to SVR12 was similar to that observed in patients with HOMA-IR ≥ 2.5 or a TyG index ≥ 8.27. Triglyceride levels showed a significant reduction (91.0 [IQR: 61.0–112.0] vs. 71.0 [IQR: 56.0–108.0] mg/dL; *p* = 0.036). Although fasting plasma glucose and HbA1c levels did not significantly improve over the short follow-up period, patients with prediabetes exhibited significant reductions in both HOMA-IR (2.9 [IQR: 1.7–4.1] vs. 2.0 [IQR: 1.4–2.7]; *p* < 0.001) and TyG index (8.44 [IQR: 8.03–8.69] vs. 8.31 [IQR: 7.94–8.65]; *p* = 0.007) following HCV eradication (Figure 4). These improvements were not observed in patients without prediabetes.

## 4. Discussion

Hepatitis C virus infection can induce insulin resistance in both the liver and peripheral tissues, making it an important prognostic factor, such as the development of HCC [3]. The eradication of HCV has been shown to improve insulin resistance; therefore, establishing an accurate and convenient indicator for determining insulin resistance after SVR12 is highly anticipated [4]. While the HOMA-IR is a widely validated tool for assessing insulin resistance, it is not routinely used in clinical practice [15]. In contrast, the TyG index, derived from fasting triglyceride and glucose levels, is a simple, accessible, and reliable surrogate marker for insulin resistance. This prospective study is the first to explore the use of the TyG index to assess insulin resistance following DAA treatment. In this cohort, the TyG index exhibited a positive correlation with HOMA-IR. Among patients with pre-existing insulin resistance, both HOMA-IR and the TyG index showed significant improvements at SVR12. Using a TyG index threshold of 8.27 to define insulin resistance, a similar pattern of improvement was observed in both HOMA-IR and the TyG index at SVR12. These findings suggest that the TyG index could serve as a valuable tool for monitoring changes in insulin resistance following HCV eradication.

The TyG index has demonstrated strong performance in estimating insulin resistance compared to HOMA-IR and has emerged as a valuable biomarker for assessing cardiovascular risk, metabolic syndrome, and related conditions [17,27]. Moreover, the TyG index provides reliable diagnostic and predictive accuracy for metabolic dysfunction-associated fatty liver disease (MAFLD) [28]. A reliable biomarker of metabolic dysfunction is particularly important for HCV-infected patients, who often continue to experience metabolic impairments, including insulin resistance and MAFLD, even after achieving virological cure. These persistent metabolic abnormalities represent critical risk factors for liver disease progression, HCC development, and the onset of extrahepatic conditions [11,29]. Our study further highlights the clinical utility of the TyG index, showing that in patients with baseline insulin resistance, changes in the TyG index were aligned with those observed in HOMA-IR. This consistency highlights the potential of the TyG index as a practical tool for designing monitoring strategies and implementing risk stratification to manage long-term complications, such as HCC, after HCV eradication.

Chronic HCV infection disrupts host metabolism by impairing glucose regulation and altering lipid metabolism. These disruptions occur as viral proteins hijack hepatocyte metabolic pathways, either directly by interfering with intracellular signaling or indirectly by promoting peripheral insulin resistance [3]. Treatment with DAA has been shown to affect serum cholesterol levels after HCV eradication, leading to increases in total cholesterol, LDL cholesterol, and HDL cholesterol [7,30]. Consistent with previous studies, our cohort exhibited similar patterns of cholesterol changes following DAA treatment. However, changes in triglyceride levels post-DAA treatment have been less consistent across prior real-world studies [30]. To ensure the reliability of observed metabolic changes, we excluded patients using anti-diabetic or lipid-lowering medications during the treatment and follow-up periods. We observed a general decline in triglyceride levels after DAA treatment. Notably, triglyceride decreased significantly among patients with pre-existing insulin resistance, which further contributed to the improvement in the TyG index. Additionally, there were significant improvements in HOMA-IR at SVR12 across the entire cohort, accompanied by a marked reduction in insulin levels. Notably, patients with pre-existing insulin resistance experienced the greatest improvements in HOMA-IR, while those without baseline insulin resistance showed minimal changes. These results suggest that the impact of DAA treatment on insulin resistance is more pronounced in patients with baseline metabolic dysfunction. The mechanisms underlying the improvement in the TyG index may be multifaceted. On one hand, the improvement is directly related to better glucose regulation, similar to HOMA-IR. On the other hand, although HCV eradication alters lipid metabolism, it does not appear to directly influence triglyceride reductions or the associated TyG index improvement. These findings reinforce the TyG index as a reliable and accessible biomarker of insulin resistance changes after HCV eradication.

Chronic insulin resistance is a key contributor to various long-term health complications, including the development of HCC and cardiovascular disease [3]. The eradication of HCV may help improve insulin resistance and thereby reduce the risk of these associated conditions [31]. However, not all individuals with insulin resistance experience metabolic improvement following HCV clearance [11]. For instance, a recent cohort study reported that patients with pre-existing diabetes were less likely to achieve steatosis regression and were at higher risk for developing new-onset steatosis after achieving SVR [32]. These findings suggest that, despite successful viral eradication, individuals with underlying insulin resistance may still be predisposed to hepatic and metabolic complications. Therefore, ongoing monitoring of the condition of insulin resistance remains crucial for predicting and promptly managing potential long-term adverse outcomes. The findings of this study support the potential utility of the TyG index as a simple, practical biomarker for assessing insulin resistance over time. However, the interpretation of specific cutoffs and clinically meaningful changes warrants further validation through larger, long-term prospective studies.

Several limitations should be acknowledged in this study. Firstly, the sample size of this study was relatively small, which may limit the generalizability of the findings. However, the significant results observed in this prospective study provide a solid foundation for future large-scale investigations. Secondly, the follow-up period in this study was relatively short, which restricts our ability to evaluate the long-term impact of HCV eradication on the TyG index, as well as its association with clinical outcomes such as cardiovascular events or HCC development. Further studies with longer-term follow-up may be beneficial for a more comprehensive understanding of the long-term effects of HCV eradication on the TyG index. Third, the present study excluded patients receiving medications for diabetes or hyperlipidemia to minimize confounding factors and ensure sample homogeneity. Further studies are needed to evaluate the applicability of the TyG index in these patient populations.

In conclusion, DAA therapy significantly improves insulin resistance, as evidenced by notable reductions in HOMA-IR and the TyG index. The TyG index may serve as a valuable tool for monitoring changes in insulin resistance following HCV eradication, particularly in patients with baseline insulin resistance.

## Figures and Tables

**Table 1:** Baseline characteristics of the study subjects.

Variables	*n* = 111	
Age, years	61.0	(55.0–73.0)
Male, *n* (%)	51	(45.9)
Hepatitis C virus RNA, log IU/mL	5.8	(5.2–6.5)
Aspartate aminotransferase, U/L	39.0	(26.0–70.0)
Alanine aminotransferase, U/L	44.0	(22.0–95.0)
APRI	0.7	(0.4–1.4)
FIB-4 index	2.5	(1.5–4.1)
<1.45, *n* (%)	26	(52.3)
1.45 to 3.25, *n* (%)	47	(47.7)
>3.25, *n* (%)	38	(52.3)
Liver cirrhosis, *n* (%)	20	(18.0)
Direct-acting antivirals		
sofosbuvir based, *n* (%)	42	(37.8)
glecaprevir + pibrentasvir, *n* (%)	38	(34.2)
elbasvir + grazoprevir, *n* (%)	12	(10.8)
ombitasvir/paritaprevir/ritonavir + dasabuvir, *n* (%)	13	(11.7)
daclatasvir + asunaprevir, *n* (%)	6	(5.4)
Coexisting hepatic steatosis, *n* (%)	69	(62.2)
Mild, *n* (%)	43	(38.7)
Moderate, *n* (%)	19	(17.1)
Severe, *n* (%)	7	(6.3)
Body mass index, kg/m^2^	23.1	(21.1–26.3)
Total cholesterol, mg/dL	161.0	(144.0–185.0)
HDL cholesterol, mg/dL	54.0	(41.0–66.0)
LDL cholesterol, mg/dL	93.0	(78.0–109.0)
Triglyceride, mg/dL	89.0	(58.0–116.0)
HbA1c, %	5.5	(5.2–5.8)
Fasting plasma glucose, mg/dL	96.0	(90.0–104.0)
Fasting plasma insulin, μU/mL	9.6	(5.7–15.1)
Prediabetes, *n* (%)	59	(53.2)
HOMA-IR score	2.4	(1.4–3.9)
HOMA-IR score < 2.5, *n* (%)	58	(52.3)
HOMA-IR score ≥ 2.5, *n* (%)	53	(47.7)
TyG index	8.36	(7.99–8.64)

Continuous variables are expressed as a median (interquartile range). APRI, aspartate aminotransferase to platelet ratio index; FIB-4 index, fibrosis-4 index; HbA1C, glycosylated hemoglobin; HDL, high-density lipoprotein; HOMA-IR, homeostasis model assessment of insulin resistance; LDL, low-density lipoprotein; RNA, ribonucleic acid; TyG index, triglyceride-glucose index.

**Table 2:** Subgroup analysis of parameter changes at SVR12 by baseline HOMA-IR score.

	HOMA-IR < 2.5 *n* = 58					HOMA-IR ≥ 2.5 *n* = 53				
	Baseline		SVR12		*p* Value	Baseline		SVR12		*p* Value
AST, U/L	41.5	(27.0–70.0)	22.5	(18.8–30.0)	<0.001	38.0	(25.5–71.5)	27.0	(20.5–38.0)	0.001
ALT, U/L	49.0	(20.8–87.0)	16.5	(13.0–22.3)	<0.001	43.0	(22.5–105.5)	21.0	(15.0–29.5)	<0.001
APRI	0.65	(0.39–1.34)	0.31	(0.23–0.68)	<0.001	0.68	(0.45–1.59)	0.41	(0.32–1.14)	<0.001
FIB-4 index	2.37	(1.50–3.63)	1.89	(1.21–3.47)	0.149	2.64	(1.46–4.30)	2.63	(1.6–5.04)	0.583
Total cholesterol, mg/dL	161.0	(148.3–185.0)	177.5	(160.5–208.0)	<0.001	158.0	(140.0–184.5)	179.0	(153.0–203.0)	0.004
HDL cholesterol, mg/dL	56.0	(41.7–66.7)	56.5	(46.9–72.5)	0.004	51.0	(40.5–64.5)	52.0	(41.4–71.0)	0.066
LDL cholesterol, mg/dL	96.0	(79.0–111.5)	109.0	(92.8–128.3)	<0.001	92.0	(75.5–108.5)	112.0	(90.5–123.5)	0.006
Triglyceride, mg/dL	82.5	(53.8–112.0)	70.0	(55.8–105.8)	0.596	101.0	(65.0–125.5)	87.0	(63.5–123.0)	0.272
HbA1c, %	5.4	(5.2–5.8)	5.6	(5.3–5.8)	0.061	5.5	(5.3–5.8)	5.5	(5.3–5.8)	1.000
Fasting plasma glucose, mg/dL	94.5	(86.8–99.0)	96.0	(91.0–101.5)	0.001	100.0	(96.0–109.0)	100.0	(90.0–109.0)	0.028
Fasting plasma insulin, μU/mL	6.2	(4.5–8.0)	5.3	(4.0–7.5)	1.000	15.4	(12.4–23.9)	10.3	(7.8–16.2)	<0.001
HOMA-IR score	1.4	(1.0–2.0)	1.3	(0.9–2.0)	0.896	4.0	(3.1–5.4)	2.5	(2.0–3.9)	<0.001
TyG index	8.20	(7.88–8.57)	8.09	(7.90–8.57)	1.000	8.47	(8.08–8.68)	8.36	(8.00–8.71)	0.028

Continuous variables are expressed as a median (interquartile range). ALT, alanine aminotransferase; APRI, aspartate aminotransferase to platelet ratio index; AST, aspartate aminotransferase; FIB-4, fibrosis-4; HbA1C, glycosylated hemoglobin; HDL, high-density lipoprotein; HOMA-IR, homeostasis model assessment index of insulin resistance; LDL, low-density lipoprotein; SVR12, sustained virological response at 12 weeks post-treatment; TyG index, triglyceride-glucose index.

**Table 3:** Subgroup analysis of parameter changes at SVR12 by baseline TyG index.

	TyG Index < 8.27 *n* = 49					TyG Index ≥ 8.27 *n* = 62				
	Baseline		SVR12		*p* Value	Baseline		SVR12		*p* Value
AST, U/L	51.0	(25.0–71.5)	23.0	(19.0–30.0)	<0.001	37.5	(27.8–70.0)	26.0	(20.0–35.8)	<0.001
ALT, U/L	50.0	(21.0–99.5)	18.0	(13.0–21.5)	<0.001	40.5	(22.8–94.3)	19.0	(13.0–29.3)	<0.001
APRI	0.75	(0.42–1.50)	0.31	(0.22–0.75)	<0.001	0.62	(0.41–1.41)	0.41	(0.29–0.95)	<0.001
FIB-4 index	2.38	(1.23–4.10)	1.75	(1.17–4.26)	0.253	2.52	(1.55–4.11)	2.64	(1.67–3.90)	0.374
Total cholesterol, mg/dL	162.0	(138.5–187.0)	178.0	(153.0–207.0)	<0.001	160.0	(146.0–184.3)	179.0	(159.8–203.0)	<0.001
HDL cholesterol, mg/dL	59.0	(46.5–70.0)	61.0	(52.0–77.8)	0.061	47.4	(35.8–63.0)	50.5	(39.8–68.5)	0.005
LDL cholesterol, mg/dL	95.0	(76.0–105.0)	106.0	(90.0–127.0)	0.004	93.0	(78.8–111.5)	111.5	(94.8–125.3)	<0.001
Triglyceride, mg/dL	55.0	(47.0–69.0)	62.0	(52.5–74.0)	0.061	112.0	(100.3–137.8)	99.5	(70.8–139.5)	0.001
HbA1c, %	5.5	(5.4–5.7)	5.5	(5.3–5.8)	0.440	5.5	(5.2–5.9)	5.6	(5.3–5.8)	0.272
Fasting plasma glucose, mg/dL	96.0	(87.0–101.0)	94.0	(87.5–102.0)	0.651	99.0	(91.5–108.0)	99.5	(93.0–109.0)	0.699
Fasting plasma insulin, μU/mL	6.8	(4.5–14.3)	6.5	(4.0–8.8)	0.041	11.3	(8.2–16.4)	9.1	(6.1–15.9)	0.010
HOMA-IR score	1.6	(1.0–3.5)	1.6	(0.9–2.2)	0.046	2.8	(2.0–4.3)	2.3	(1.5–3.8)	0.031
TyG index	7.95	(7.69–8.12)	7.97	(7.79–8.24)	0.312	8.62	(8.46–8.83)	8.52	(8.27–8.89)	0.003

Continuous variables are expressed as a median (interquartile range). ALT, alanine aminotransferase; APRI, aspartate-aminotransferase-to-platelet ratio index; AST, aspartate aminotransferase; FIB-4, fibrosis-4; HbA1C, glycosylated hemoglobin; HDL, high-density lipoprotein; HOMA-IR, homeostasis model assessment of insulin resistance; LDL, low-density lipoprotein; SVR12, sustained virological response at 12 weeks post-treatment; TyG index, triglyceride-glucose index.

**Table 4:** Subgroup analysis of parameter changes at SVR12 in patients with prediabetes.

	Patients Without Prediabetes *n* = 52					Patients with Prediabetes *n* = 59				
	Baseline		SVR12		*p* Value	Baseline		SVR12		*p* Value
AST, U/L	43.0	(27.0–69.3)	23.5	(19.3–32.8)	<0.001	38.0	(26.0–73.0)	24.0	(20.0–31.0)	<0.001
ALT, U/L	49.5	(24.0–97.5)	19.0	(13.0–28.5)	<0.001	40.0	(21.0–95.0)	17.0	(13.0–23.0)	<0.001
APRI	0.67	(0.42–1.39)	0.36	(0.24–0.94)	<0.001	0.66	(0.41–1.49)	0.39	(0.26–0.81)	<0.001
FIB-4 index	2.41	(1.34–4.02)	2.27	(1.19–4.24)	0.278	2.63	(1.56–4.26)	2.27	(1.44–4.07)	0.127
Total cholesterol, mg/dL	160.5	(141.0–183.3)	178.0	(162.5–203.0)	<0.001	163.0	(146.0–189.0)	180.0	(154.0–206.0)	<0.001
HDL cholesterol, mg/dL	51.5	(39.3–66.5)	55.5	(40.9–73.0)	0.001	56.0	(42.0–66.0)	56.0	(47.2–71.0)	0.010
LDL cholesterol, mg/dL	91.5	(79.0–105.8)	110.5	(93.0–124.5)	<0.001	94.0	(78.0–111.0)	109.0	(91.0–127.0)	<0.001
Triglyceride, mg/dL	89.0	(53.3–116.8)	81.0	(65.3–125.0)	0.629	91.0	(61.0–112.0)	71.0	(56.0–108.0)	0.036
HbA1c, %	5.2	(5.1–5.4)	5.4	(5.1–5.6)	0.014	5.7	(5.5–6.0)	5.8	(5.5–5.9)	0.456
Fasting plasma glucose, mg/dL	92.0	(86.0–96.0)	92.0	(86.0–98.0)	0.189	103.0	(96.0–111.0)	103.0	(94.0–110.0)	0.448
Fasting plasma insulin, μU/mL	8.1	(5.5–16.0)	7.5	(4.5–11.6)	0.131	11.2	(7.0–14.7)	7.7	(5.3–11.4)	<0.001
HOMA-IR score	1.8	(1.2–3.5)	1.8	(1.0–2.8)	0.216	2.9	(1.7–4.1)	2.0	(1.4–2.7)	<0.001
TyG index	8.26	(7.80–8.56)	8.31	(7.94–8.69)	0.133	8.44	(8.03–8.69)	8.31	(7.94–8.62)	0.007

Continuous variables are expressed as a median (interquartile range). ALT, alanine aminotransferase; APRI, aspartate-aminotransferase-to-platelet ratio index; AST, aspartate aminotransferase; FIB-4, fibrosis-4; HbA1C, glycosylated hemoglobin; HDL, high-density lipoprotein; HOMA-IR, homeostasis model assessment of insulin resistance; LDL, low-density lipoprotein; SVR12, sustained virological response at 12 weeks post-treatment; TyG index, triglyceride-glucose index.

## Data Availability

Data are available upon the author’s request.

## Supplementary Materials

The following supporting information can be downloaded at: https://www.mdpi.com/article/10.3390/jcm14092963/s1, Figure S1: The receiver operating characteristic curve showing the optimal cut point of the TyG index compared with the HOMA-IR index for estimating insulin resistance; Figure S2: A significant correlation between the baseline HOMA-IR and the change in HOMA-IR at SVR 12; Figure S3: A significant correlation between the baseline TyG index and its change at SVR 12.
