# Supplementary material for: The Triglyceride-Glucose Index as a Biomarker for Insulin Resistance Following Hepatitis C Virus Eradication: A Prospective Cohort Study
Source: J Clin Med. 2025 Apr 25;14(9):2963. doi: 10.3390/jcm14092963 (PMC12072629; doi:10.3390/jcm14092963)
Supplement: Supplementary file 1 [file jcm-14-02963-s001.zip › jcm-3561590-supplementary.pdf]

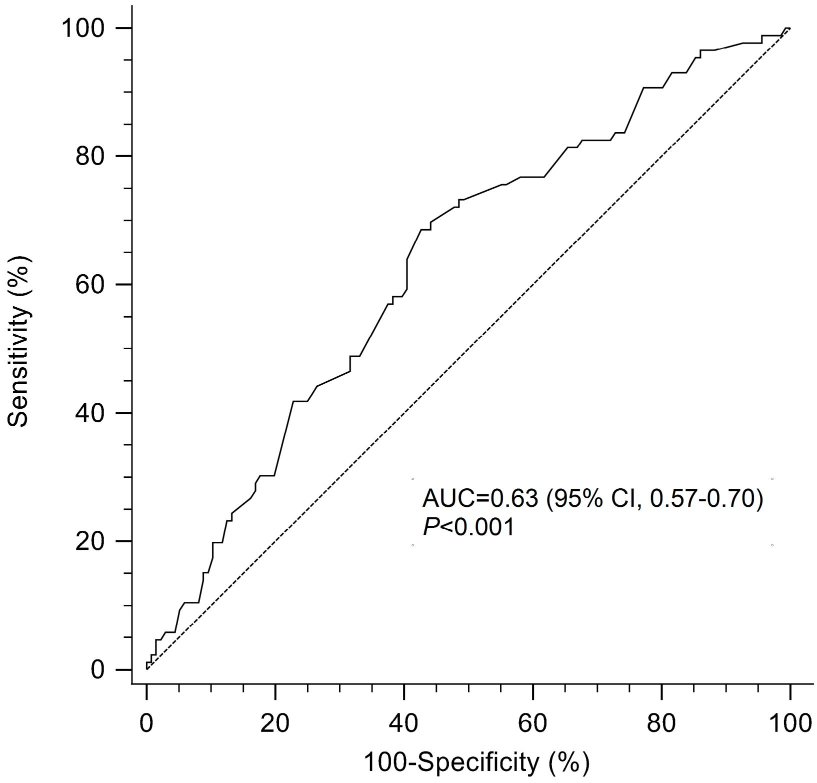

Supplemental figure S1. The receiver operating characteristic curve showing the optimal cut point of the TyG index compared with the HOMA-IR index for estimating insulin resistance. The best cut point of the TyG index was 8.27 (sensitivity 68.6% and specificity 57.4%). AUC, area under the receiver operating characteristic curve; HOMA-IR, homeostasis model assessment index for insulin resistance; TyG index, triglyceride-glucose index.

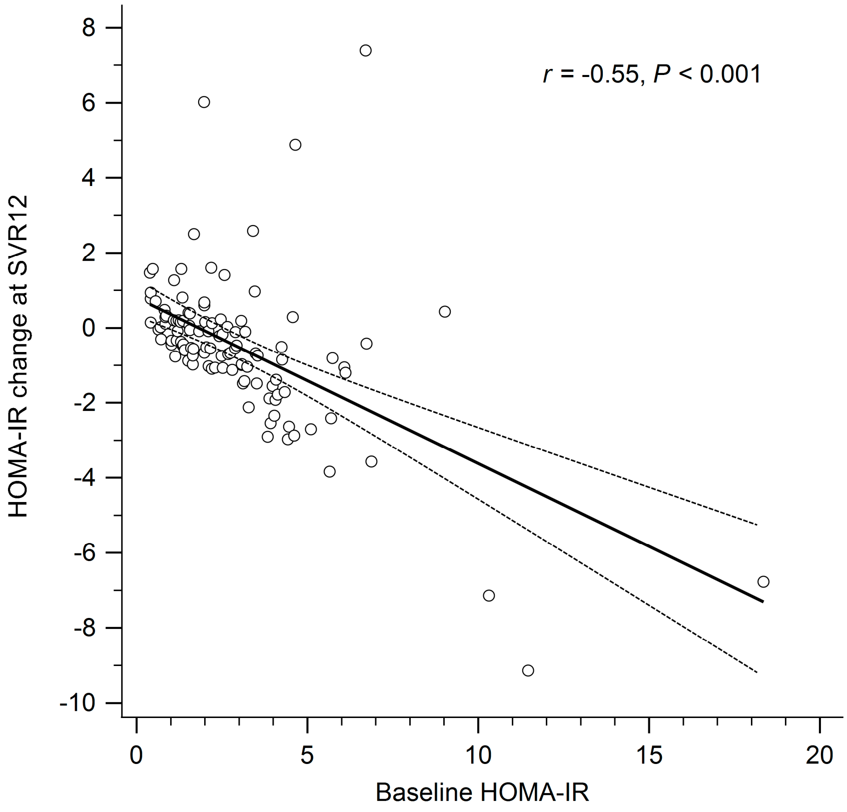

Supplemental figure S2. A significant correlation between the baseline HOMA-IR and the change in HOMA-IR at SVR 12 (Pearson's correlation coefficient, r = -0.55; P < 0.001). HOMA-IR, homeostasis model assessment index for insulin resistance; SVR12, sustained virological response at 12 weeks post-treatment.

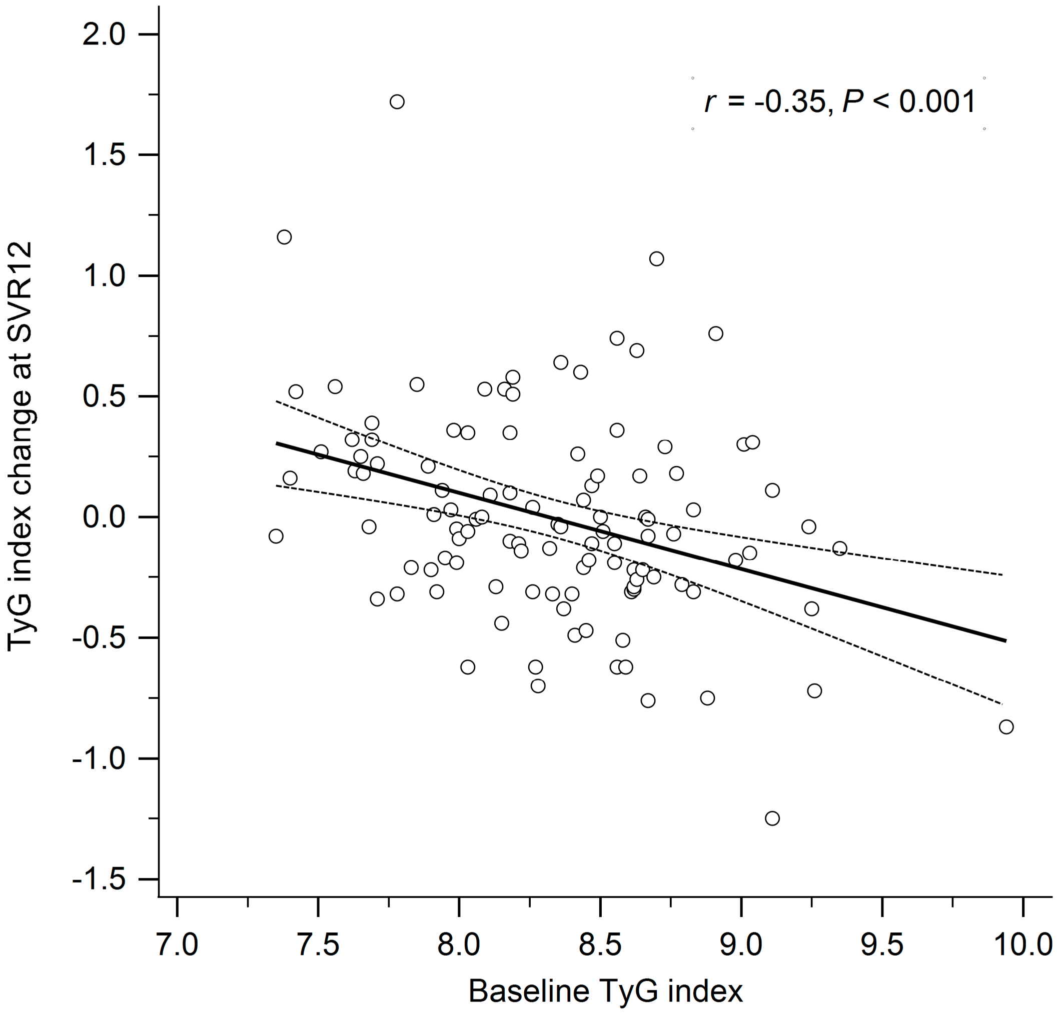

Supplemental figure S3. A significant correlation between the baseline TyG index and its change at SVR 12 (Pearson's correlation coefficient, $r = -0.35$; $P < 0.001$). SVR12, sustained virological response at 12 weeks post-treatment; TyG index, triglyceride glucose index.
